# Seroprevalence of H7N9 infection among humans: A systematic review and meta‐analysis

**DOI:** 10.1111/irv.12736

**Published:** 2020-03-10

**Authors:** Qiang Wang, Ke Xu, Weihua Xie, Liuqing Yang, Haiyan Chen, Naiyang Shi, Changjun Bao, Haodi Huang, Xuefeng Zhang, Yilan Liao, Hui Jin

**Affiliations:** ^1^ Department of Epidemiology and Health Statistics School of Public Health Southeast University Nanjing China; ^2^ Key Laboratory of Environmental Medicine Engineering Ministry of Education School of Public Health Southeast University Nanjing China; ^3^ Jiangsu Provincial Center for Disease Control and Prevention Nanjing China; ^4^ Department of Laboratory Medicine The First Affiliated Hospital Nanjing Medical University Nanjing China; ^5^ The State Key Laboratory of Resources and Environmental Information System Institute of Geographic Sciences and Natural Resources Research Chinese Academy of Sciences Beijing China

**Keywords:** H7N9, influenza A, meta‐analysis, seroprevalence, systematic review

## Abstract

In spring 2013, a novel avian‐origin influenza A (H7N9) virus emerged in mainland China. The burden of H7N9 infection was estimated based on systematic review and meta‐analysis. The systematic search for available literature was conducted using Chinese and English databases. We calculated the pooled seroprevalence of H7N9 infection and its 95% confidence interval by using Freeman‐Tukey double arcsine transformation. Out of 16 890 records found using Chinese and English databases, 54 articles were included in the meta‐analysis. These included studies of a total of 64 107 individuals. The pooled seroprevalence of H7N9 infection among humans was 0.122% (95% CI: 0.023, 0.275). In high‐risk populations, the highest pooled seroprevalence was observed among close contacts (1.075%, 95% CI: 0.000, 4.357). The seroprevalence among general population was (0.077%, 95% CI: 0.011, 0.180). Our study discovered that asymptomatic infection of H7N9 virus did occur, even if the seroprevalence among humans was low.

## 1 INTRODUCTION

In February and March 2013, a novel avian‐origin influenza A (H7N9) virus was identified, which caused more than 100 human cases in mainland China.^1^, ^2^ Up to September 5, 2018, a total of 1567 H7N9 human cases were reported, including more than 615 mortalities.^3^ The case fatality rate of H7N9 patients was close to 40%.^3^, ^4^ In March 2019, the cases of H7N9 infection re‐emerged in China after a period of 14 months.^5^ The majority of the H7N9 patients lived in mainland China. Hong Kong, Taiwan, Malaysia, and Canada also reported human cases of sporadic H7N9 infection, which were imported from mainland China.^4^

The patients with H7N9 infection who presented with severe clinical symptoms and showed a high case fatality rate have attracted global attention. The proportion of the population with mild or asymptomatic infection, that is, the iceberg below the sea level, was also worthy of attention. A number of serologic studies have been conducted on the seroprevalence of H7N9 infection among humans. However, it is difficult to estimate the extent of infection owing to different variables, such as study populations, test methods, and positive cutoff values in various studies. The purpose of our meta‐analysis was to examine the seroprevalence of influenza A (H7N9) infection among humans and to estimate the overall burden of H7N9 infection.

## 2 METHOD

### 2.1 Search strategy

A systematic search of the relevant literature was conducted for relevant articles published before October 22, 2018. The search terms were as follows: [“H7N9” OR “influenza A” OR “influenza A virus”] AND [“seroprevalence” OR “seropositive” OR “seronegative” OR “serologic” OR “serological” OR “seroepidemiology”]. Both Chinese and English databases were searched. Chinese databases consisted of the China National Knowledge Infrastructure, Chinese Science and Technology Periodical Database (VIP), and WanFang Database. English databases consisted of PubMed, Web of Science, and the Cochrane Library. Additionally, we searched the World Health Organization (WHO)'s website, regional health department's website, and reference lists of selected studies.

### 2.2 Inclusion and exclusion criteria

The inclusion criteria were as follows: (a) studies reporting the seroprevalence of H7N9 infection among humans and (b) cross‐sectional, retrospective, and cohort studies or routine surveillance.

The exclusion criteria were as follows: (a) study only examined the H7 subtype, (b) non‐H7N9 virus strains used in experiments, (c) study subjects were H7N9 patients or influenza patients, (d) sample size was too small (N < 10), (e) duplicated data, (f) study did not provide key data, that is, one‐third of the data (total number, the number of seropositive cases, and seroprevalence) were missing, and (g) conference papers.

Our aim was to summarize the antibody level against the H7N9 subtype. We excluded the serological studies that only examined H7 sole subtype or used non‐H7N9 virus strains in the test for the following reasons. First, the results of the tests only for the H7 subtype could only indicate previous infection with the H7 subtype, such as H7N1. Second, the hemagglutinin gene fragment of the influenza virus is constantly mutating. The H7 antigen of other subtypes is different from the H7N9 antigen reported in China because the H7 antigen can change.^6^, ^7^ Therefore, the results of the detection of other subtypes of H7 were not convincing. Although previous studies have shown cross‐reactivities between H7N9 and divergent H7 subtypic viruses,^8^, ^9^ the specific avian influenza A(H7N9) virus should be used in hemagglutinin inhibition (HI) or microneutralization (MN) assays.^10^

### 2.3 Quality assessment and data abstraction

Two researchers (QW and KX) independently reviewed and assessed each included article according to the following 10 criteria^11^: (a) whether it was a population‐based study, (b) whether the study time and location were provided, (c) whether the study population was ≥100 subjects, (d) whether the study population had avian exposure, (e) whether the characteristics of the study population were mentioned, (f) whether HI was carried out, (g) whether MN was carried out, (h) whether horse red blood cells were used in the HI assay, (i) whether the seropositive cutoff value was mentioned in the study, and (j) whether the seropositive cutoff value provided in the study referred to the WHO criteria. If the two researchers were in disagreement about the quality of a study, a third researcher (HJ) would make the final decision. “Yes” indicated a score of one, and “No” or “Not provided” indicated a score of zero; finally, we calculated the total score of the 10 items.

Similarly, data abstraction was carried out by two researchers (QW and KX). After extraction, data were checked by a third researcher (HJ). If there was a difference, the original literature would be reviewed for re‐extraction. The following data were extracted: first author, publication year, study type, population sample, study region, fieldwork dates, sample size, number of seropositive cases, seroprevalence, test method, seropositive cutoff value, HI test cell, and number of humans in each dilution titer (1:10‐1:640). For cohort studies, the number of people who showed seroconversion, the criteria of seroconversion, and follow‐up time was also extracted.

### 2.4 Data analysis

Excel and Stata software were used in this study. The data were subjected to Freeman‐Tukey double arcsine transformation, and we reported the pooled seroprevalence and its 95% confidence interval (CI) using the DerSimonian‐Laird random effects.^12^, ^13^ Analyses were conducted using the metaprop package in Stata software.^14^ We assessed the heterogeneity between the studies with the *I* ^2^ statistic. If the heterogeneity test result was *I* ^2^ < 50%, a fixed effect model was used; otherwise, a random effect model was used.

The WHO has suggested the criteria for confirming whether the results of H7N9 serologic tests are positive: for single‐serum samples, HI ≥ 1:160; for paired serum samples (acute and convalescent sera), a 4‐fold rise in HI titer.^15^ In single‐serum samples, sera with HI titer of 20‐80 should be confirmed by MN or WB assay.^15^ In addition to pooling seroprevalence according to the original study criteria, we re‐judged the seropositive results according to the WHO criteria to explore the influence of different thresholds on pooled seroprevalence.^11^ Based on the studies that reported the number of humans in each titer, we re‐judged the seropositive results in the included studies. The statistical significance of H7N9 seroprevalences that were calculated by the WHO criteria and original study criteria was assessed using the Wilcoxon rank sum test. We performed statistical tests for the included studies that provided number of humans in each titer to ensure comparability. For cohort studies, the incidence of seroconversion was analyzed after calculating data using the same standard unit (per person‐months), defined as follows: number of seroconverted humans in the cohorts divided by the number of person‐months of follow‐up. We further performed stratified subgroup and meta‐regression analyses.

## 3 RESULTS

### 3.1 Search results

**Figure 1:**
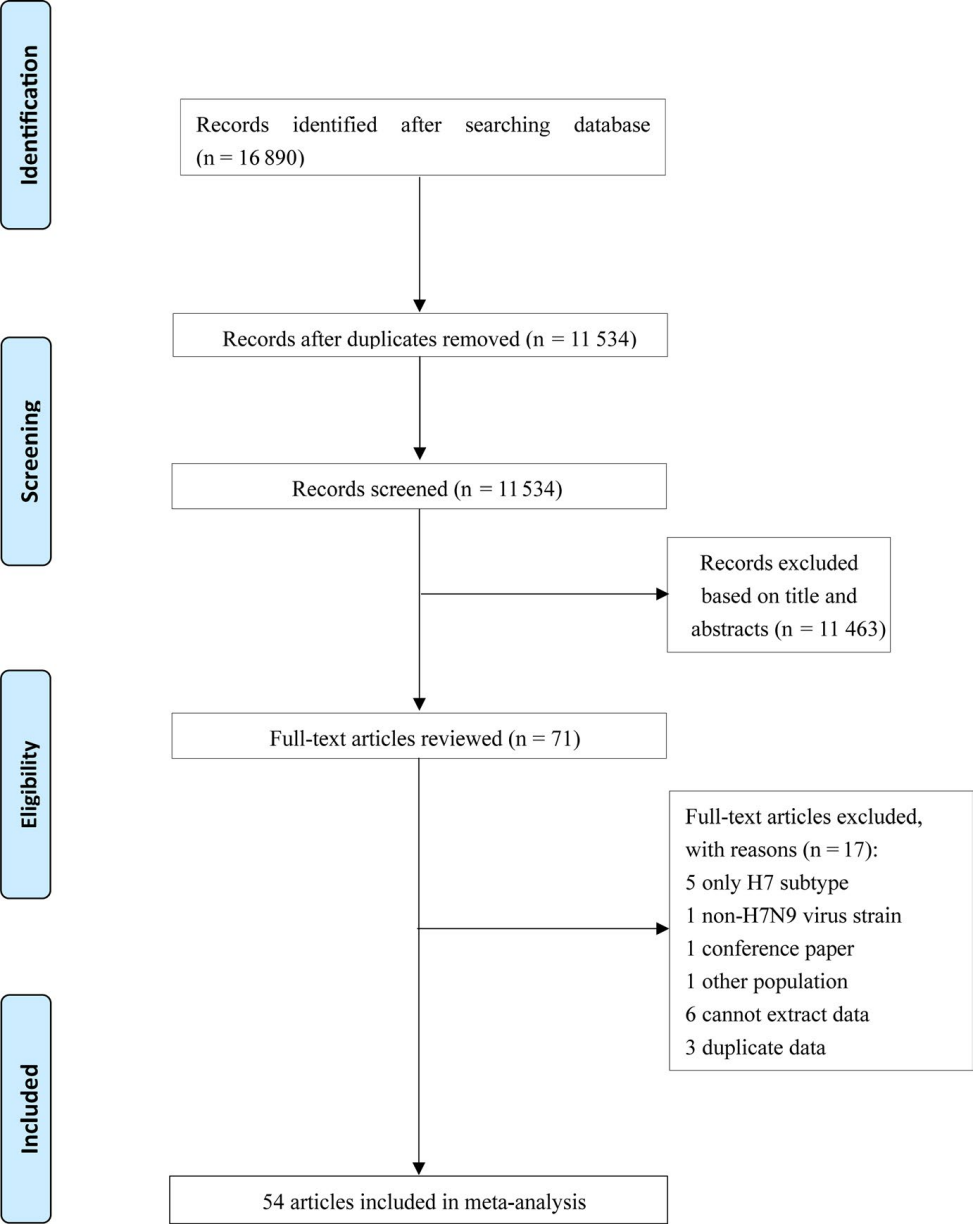
Flowchart of the literature search and study selection

A total of 16 890 records were obtained from Chinese and English databases according to the search terms mentioned before, of which 71 articles were reviewed in full‐text (Figure 1). Further, 17 articles were excluded on the basis of the exclusion criteria: 5 studies only examined H7 sole subtype, 1 study used other H7 subtype in the test, 1 study involved H7N9 patients who survived, 6 studies did not provide the data, 3 studies provided replicated data, and 1 study was a conference study. Finally, 54 studies were included in the analysis, consisting of a total of 64 107 individuals.

### 3.2 Study characteristics and quality assessment

The 54 included studies were conducted in different regions and with different populations (Table S1). One study was from Taiwan, one from Hong Kong, and the rest were from mainland China. One study conducted in India was excluded because a non‐H7N9 virus strain was used in the experiments,^16^ and one study in Vietnam was excluded because it only examined the H7 subtype.^17^ There were 43 articles that involved poultry workers, 5 articles that involved swine workers, 8 articles that involved close contacts, and 14 articles that involved the general population. Some articles provided data on various study populations.

Of the 54 studies, 3 studies reported the seroprevalence before 2013, 13 studies reported it during the first epidemic wave (1/2013‐9/2013), 14 studies during the second epidemic wave (10/2013‐9/2014), and 4 studies during the third epidemic wave (10/2014‐9/2015). The other studies did not provide the study period or provided ambiguous dates that were difficult to classify; 23 studies followed the WHO criteria, 21 studies did not, and others did not specify the criteria. The scores of quality assessment ranged from 3 to 10 points, with an average of 7.2 ± 1.8 (Table S2).

### 3.3 Seroprevalence of influenza A (H7N9)

The pooled seroprevalence of H7N9 infection among humans was 0.122% (95% CI: 0.023, 0.275). The seroprevalence reported in the included studies ranged from 0.000% to 17.143%. In the 37 included studies that reported the number of people in each titer, the pooled seroprevalence was 0.046% (95% CI: 0.000, 0.193) according to the original study criteria, but was 0.003% (95% CI: 0.000, 0.081) according to the WHO criteria. However, the difference was not statistically significant (*Z* = −1.334, *P* = .182). Of the 37 articles, 14 were not in accordance with the WHO criteria. The pooled incidence of seroconversion in our study was 0.087% (95% CI: 0.007, 0.223) per person‐months.

**Figure 2:**
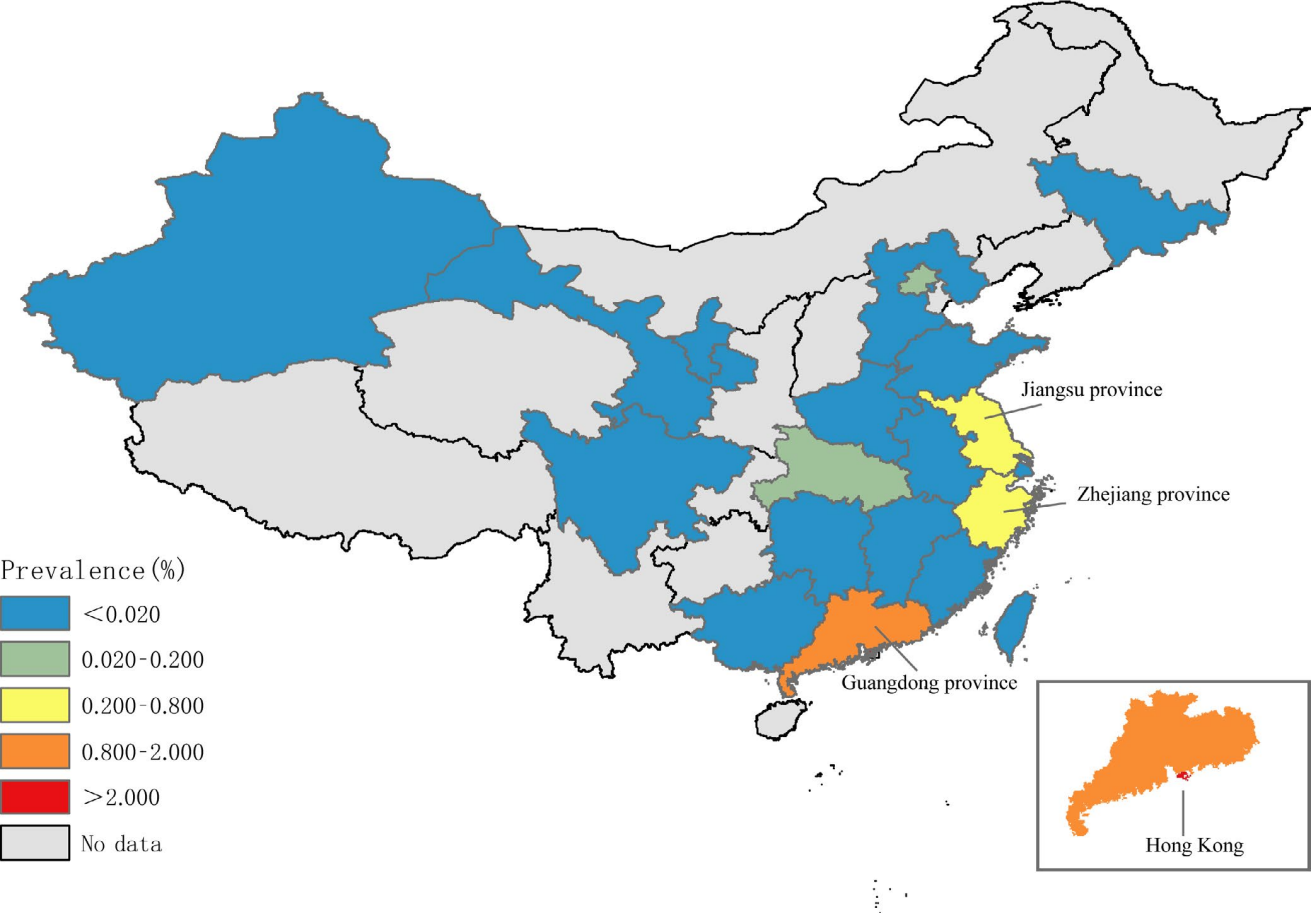
Seroprevalence of H7N9 virus among humans

The seroprevalence of the H7N9 virus varied widely in different regions (Table 1, Figure 2). We reported the seroprevalence in different regions of mainland China, including the eastern, central, and western regions.^18^ The seroprevalence in the eastern region was higher than that in the other two regions.

**Table 1:** Seroprevalence in different groups^a^

Variable		Ref (n)	n	Event	Seroprevalence (%)	95% CI (%)	*I* ^2^ (%)
Total		54	64 107	410	0.122	0.023, 0.275	88.6
Different populations	Poultry workers	43	27 383	317	0.254	0.041, 0.584	90.2
	Swine workers	5	8596	5	0.005	0.000, 0.064	19.6
	Close contacts	8	793	21	1.075	0.000, 4.357	74.7
	General population	14	25 620	50	0.077	0.011, 0.180	71.1
Time	Before 2013	3	3089	0	0.000	0.000, 0.053	0.0
	First epidemic wave	13	10 166	95	0.109	0.000, 0.670	90.3
	Second epidemic wave	14	22 550	166	0.441	0.101, 0.942	93.1
	Third epidemic wave	4	6005	4	0.000	0.000, 0.000	53.6
Region in mainland China	Eastern	34	52 458	389	0.129	0.009, 0.346	92.0
	Central	9	6819	8	0.000	0.000, 0.000	8.6
	Western	12	4514	0	0.000	0.000, 0.006	0.0
Seropositive value	HI titer ≥ 1:20	6	4942	13	0.007	0.000, 0.226	66.2
	HI titer ≥ 1:40	5	9200	93	1.056	0.247, 2.348	95.5
	HI titer ≥ 1:80	7	18 698	94	0.147	0.001, 0.440	90.1
	HI titer ≥ 1:160	18	7971	146	0.500	0.003, 1.501	92.8
	HI titer ≥ 1:20 and MN titer ≥ 1:20	4	6975	13	0.000	0.000, 0.033	40.1
Test HI cell	Turkey red blood cell	7	4910	33	0.013	0.000, 0.506	82.6
	Horse red blood cell	36	47 961	303	0.158	0.026, 0.364	90.1
	Chicken red blood cell	4	3781	10	0.000	0.000, 0.229	66.5
Test method	HI	40	39 263	329	0.203	0.026, 0.490	91.6
	HI an MN	11	23 114	81	0.078	0.000, 0.245	79.3
Sample size	＜500	46	18 659	212	0.169	0.005, 0.484	86.1
	≥500	19	45 317	198	0.213	0.078, 0.398	91.2

^a^ HI: Hemagglutination inhibition test; MN: Microneutralization test.

The seroprevalence among close contacts was 1.075% (95% CI: 0.000, 4.357), ranging from 0.000% to 14.286%. The seroprevalence among close contacts was the highest in Jiangsu province (Table S3). The seroprevalence rates among poultry and swine workers were 0.254% (95% CI: 0.041, 0.584) and 0.005% (95% CI: 0.000, 0.064), respectively. The seroprevalence among poultry workers was the highest in Hong Kong and the lowest in some central and western provinces in mainland China. The seroprevalence among humans before 2013 was 0.000%. The seroprevalence was higher in the first two epidemic waves than in the third one.

### 3.4 Meta‐analysis regression

The results of univariate analysis showed that time and population significantly affected the heterogeneity of the meta‐analysis results (Table 2). We added region to the multivariate analysis because its adjusted *R* ^2^ was 2.55% in the univariate analysis. The results showed that the variables included in the regression were time and population, and the adjusted *R* ^2^ was 15.89%, which suggested that time and population can explain part of the heterogeneity.

**Table 2:** The results of meta‐regression^a^

Covariate	Coefficient	95% CI	*t*	*P*	Adjusted *R* ^2^ (%)
Univariate analysis					
Time					
Other time	‐	‐	‐	‐	4.25
First and second epidemic wave	0.068	0.009, 1.126	2.27	.025	
Region in mainland China					
Western	‐	‐	‐	‐	2.55
Eastern	0.084	−0.017, 0.185	1.65	.102	
Central	0.017	−0.095, 0.129	0.30	.763	
Population					
Swine worker	‐	‐	‐	‐	4.24
Close contacts	0.220	0.054, 0.385	2.63	.010	
Poultry workers	0.103	0.006, 0.200	2.09	.038	
General population	0.024	−0.082, 0.130	0.44	.657	
Test method					
HI and MN	‐	‐	‐	‐	−0.15
HI	0.039	−0.026, 0.103	1.19	.237	
Test HI cell					
Chicken red blood cell	‐	‐	‐	‐	−2.24
Turkey red blood cell	0.011	−0.148, 0.169	0.13	.895	
Horse red blood cell	0.016	−0.112, 0.144	0.25	.805	
Sample size					
n ≥ 500	‐	‐	‐	‐	0.06
n < 500	0.039	−0.020, 0.098	1.30	.197	
Multivariate analysis					15.89
Time					
Other time	‐	‐	‐	‐	‐
First and second epidemic wave	0.062	0.005, 0.119	2.17	.032	
Region in mainland China					
Western	‐	‐	‐	‐	‐
Eastern	0.099	−0.002, 0.201	1.94	.055	
Central	0.022	−0.088, 0.133	0.40	.693	
Population					
Swine worker	‐	‐	‐	‐	‐
Close contacts	0.152	−0.027, 0.332	1.68	.095	
Poultry workers	0.151	0.057, 0.245	3.18	.002	
General population	0.050	−0.050, 0.150	0.98	.327	

^a^ “‐”: the first line of every covariate represented reference; adjusted *R* ^2^ was used to indicate the degree of heterogeneity explained by study characteristics.

## 4 DISCUSSION

We performed this systematic review and meta‐analysis of the serological studies on influenza A (H7N9) to estimate the burden of this virus among humans. Strikingly, mild or asymptomatic human infection with H7N9 did exist, even if the proportion was small. Similar to influenza A (H5N1 and H9N2), the reported human cases were only a tip of the iceberg of a large number of infections.^11^, ^19^

High seroprevalence among poultry workers suggests that avian exposure is a risk factor for infection. A previous study on H5N1 and H9N2 infections suggested that age and chronic lung problems were consistent with elevated titers.^20^ Some case‐control studies on clinical H7N9 patients also found that patients with chronic obstructive lung disease (COPD) and those receiving immunosuppressive medications were highly susceptible to be infected with influenza virus infection.^21^, ^22^ Hence, avian exposure, chronic lung problems, and poor immune status may be associated with influenza A (H7N9) infection, in both clinical H7N9 cases and silent infections.

Poultry workers with intense avian exposure tend to have high risk of being infected with the influenza virus.^20^ However, the data were consistent with the same phenomenon seen with H5N1, where the virus was so poorly adapted to humans that most hosts could not be productively infected, leading to high exposure to the virus but low seroprevalence.^23^ Previous studies have provided evidence that the host gene plays an important role in susceptibility to infection and clinical outcomes.^24^, ^25^ The severe influenza cases are the result of rare genetic susceptibilities, attributable to the interferon‐induced transmembrane protein 3 (IFITM3) or other risk factors.^23^, ^26^, ^27^, ^28^ The possible association between glycine decarboxylase (GLDC), IFITM3, and toll‐like receptors 3 (TLR3) and the outcomes of influenza A (H7N9) infection was also examined by a few researchers.^29^, ^30^ Controversially, an epidemiological study analyzing clusters of H7N9 patients found that genetic susceptibility to H7N9 virus infection was limited.^31^ The association between susceptibility‐conferring genes and influenza A (H7N9) virus infection warrants further in‐depth studies.

The risk of infection among humans was high as per the pooled seroprevalence of close contacts. Close contacts were defined as healthcare workers who had not taken effective protective measures and family members who took care of patients during the treatment of suspected or confirmed infection; the staff who lived with the patients or had experienced other close contact situations from one day before the suspicion or confirmation of infection were placed in isolation.^32^ Articles providing information about close contacts were reviewed for further evidence. Of the 8 studies that reported the data, the seroprevalence in 5 studies was 0.000%; in the remaining 3 studies, it was 3.200%, 6.667%, and 14.286%. In the three studies that reported seropositivity, only one study reported that none were exposed to poultry, swine, or other animals.^33^ The seropositive close contacts in the study included healthcare workers and family members.^33^ Previous studies suggested that the transmissibility of H7N9 virus among persons cannot be ignored, even if it was limited.^34^, ^35^, ^36^ The close contacts of H7N9 patients require protective measures and more close attention.

Swine, the intermediate hosts that facilitate the reassortment of influenza viruses, were likely to cause infection in humans.^37^, ^38^ The animal could provide a suitable vessel for the influenza A (H7N9) virus to survive and evolve.^39^ Controversially, the seroprevalence of H7N9 among swine workers was low in our pooled results. On one hand, this might be associated with swine infected with H7N9. The previous serological reports did not provide evidence of swine involvement in H7N9 virus ecology.^40^, ^41^, ^42^ One study had shown that the H7N9 virus might not efficiently infect swine.^43^ On the other hand, the virus transmission ability from pigs to other mammals might be limited.^44^ The reasons mentioned above may explain the low seroprevalence among swine workers. More studies need to explore the difference between the adaption of H7N9 and other influenza A subtypes to swine.

With regard to time, cases of subclinical H7N9 infection were not reported before 2013. Compared with the first epidemic wave, mild or asymptomatic infection showed a downward trend during the third epidemic wave. The small number of studies on seroprevalence during this wave might have led to this finding. Besides, studies showed that antibody titers in people waned over time.^45^, ^46^ China experienced six waves of H7N9 epidemics.^3^ The highest number of humans cases was reported in the fifth epidemic wave.^47^ However, only three laboratory‐confirmed human cases were reported in the recent wave.^3^ The situation of asymptomatic infection with the H7N9 virus is unclear. Studies on the seroprevalence of the H7N9 virus among humans need to be performed, especially considering the sixth wave when cases of H7N9 infection were rarely reported.

Of note, there were some limitations to our study. First, cross‐sectional studies accounted for most of the included studies. The proportion rather than incidence was provided in these studies. The serologic results of a cross‐sectional study can be misleading because antibodies may wane over time. The risk factors for infection could not be explored exhaustively because the included studies provided inadequate information about the demographic, health, and exposure variables. Second, the different seropositive thresholds might have led to the overestimation of the seroprevalence among humans. Third, cross‐reactive immunity cannot be ignored despite excluding those articles. Fourth, the result of the meta‐regression did not explain the source of heterogeneity. Probably, the proportion was too small or even zero in most of the included studies.

## 5 CONCLUSION

Our study found that subclinical infection with the H7N9 virus did occur, even if the seroprevalence among humans was low. Be it the high‐risk group or general population, a certain degree of infection did exist. Stringent seropositive standards should be developed and observed to ensure that serology assays are reliable and convincing. Sensitive detection tests for the influenza A (H7N9) virus are need to be carried out to provide warnings before the evolvement and adaptation of the virus to the human body.

## CONFLICT OF INTEREST

The authors declare that they have no competing interests.

## AUTHOR CONTRIBUTIONS

QW and HJ designed the study. QW, KX, LQY, and WHX conducted the literature search and review. QW and KX reviewed citations and extracted data. QW, NYS, HYC, HDH, CJB, XFZ, and YLL analyzed the data. HJ and QW interpreted the results. All authors critically revised for important intellectual content. All authors approved the final version. **Qiang Wang**: Conceptualization (equal); data curation (lead); formal analysis (lead); funding acquisition (equal); methodology (equal); writing‐review & editing (lead). **Ke Xu**: Data curation (equal); formal analysis (equal); methodology (equal). **Weihua Xie**: Data curation (equal); methodology (equal). **Liuqing Yang**: Data curation (equal); methodology (equal). **Haiyan Chen**: Methodology‐Supporting. **Naiyang Shi**: Data curation (equal); methodology (equal). **Changjun Bao**: Formal analysis (equal); supervision (equal). **Haodi Huang**: Formal analysis (equal). **Xuefeng Zhang**: Formal analysis (equal); supervision (equal). **Yilan Liao**: Formal analysis (equal). **Hui Jin**: Conceptualization (equal); methodology (equal); supervision (lead); writing‐review & editing (equal).

## Supporting information

Table S1

Table S2

Table S3
