# Supplementary material for: Seroprevalence of H7N9 infection among humans: A systematic review and meta‐analysis
Source: Influenza Other Respir Viruses. 2020 Mar 10;14(5):587–95. doi: 10.1111/irv.12736 (PMC7431636; doi:10.1111/irv.12736)
Supplement: Supplementary file 1 — Table S1 [file IRV-14-587-s001.docx]

Table S1 Characteristics of included 54 studies*

| **Author** | **Publication year** | **Study type** | **Location** | **Survey time** | **Study population** | **Test method** | **HI test cell** | **Seropositive value** | **Ref** |
| --- | --- | --- | --- | --- | --- | --- | --- | --- | --- |
| Bai | 2013 | R Survey | Mainland China | Before 2013 | Poultry workers | HI and MN | Turkey RBC | HI titer ≥ 1:20 and MN titer ≥ 1:20 | 1 |
| Szu-Min | 2013 | CS Survey | Taiwan | - | Close contacts | HI | Turkey RBC | - | 2 |
| Qi | 2013 | CS Survey | Mainland China | First epidemic wave | Close contacts | HI | Turkey RBC | - | 3 |
| Xu | 2013 | CS Survey | Mainland China | First epidemic wave | Healthcare workers | HI | Pig RBC | HI titer ≥ 1:20 | 4 |
| Chen | 2014 | CS Survey | Mainland China | Second epidemic wave | Poultry workers | HI | Horse RBC | HI titer ≥ 1:40 | 5 |
| Wang | 2014 | P Cohort | Mainland China | First and second epidemic wave | Poultry workers and general population | HI | Horse RBC | HI titer ≥ 1:160 | 6 |
| Wu | 2014 | CS Survey | Mainland China | First epidemic wave | Close contacts, poultry workers, exposed to infected poultry, and exposed to contaminated environment | HI and MN | Horse RBC | HI titer ≥ 1:40 | 7 |
| Xiong | 2014 | CS Survey | Mainland China | - | General population | HI | Chicken RBC | HI titer ≥ 1:80 | 8 |
| Yang | 2014 | CS Survey | Mainland China | First epidemic wave | general population, and poultry workers | HI | Turkey RBC | HI titer ≥ 1:80 | 9 |
| Zhou | 2014 | CS Survey | Mainland China | First epidemic wave | veterinarians | HI | Horse RBC | HI titer ≥ 1:20 | 10 |
| Lu | 2014 | CS Survey | Mainland China | First epidemic wave | Poultry workers | HI | Chicken RBC | HI titer ≥ 1:20 | 11 |
| Luo | 2014 | CS Survey | Mainland China | First epidemic wave | Close contacts | HI | Turkey RBC | - | 12 |
| Xia | 2014 | CS Survey | Mainland China | First epidemic wave | General population and poultry workers | HI and MN | Horse RBC | HI titer ≥ 1:20 and MN titer ≥ 1:20 | 13 |
| Chen | 2015 | CS Survey | Mainland China | Second epidemic wave | Animal-exposed participants and general population | HI | Horse RBC | HI titer ≥ 1:40 | 14 |
| Fang | 2015 | CS Survey | Mainland China | Third epidemic wave | Close contacts | HI and MN | Horse RBC | - | 15 |
| Fan | 2015 | CS Survey | Mainland China | First and second epidemic wave | Poultry workers | HI | Horse RBC | - | 16 |
| Ma | 2015 | CS Survey | Mainland China | - | Close contacts | HI and MN | Horse RBC | HI titer ≥ 1:40 | 17 |
| Yin | 2015 | R Survey | Mainland China | Before 2013 | Veterinarians | ELISA and HI | Horse RBC | HI titer ≥ 1:80 | 18 |
| Chen | 2015 | CS Survey | Mainland China | Second epidemic wave | Poultry workers and swine workers | - | - | HI titer ≥ 1:160 | 19 |
| Lu | 2015 | CS Survey | Mainland China | Second epidemic wave | General population | HI | - | HI titer ≥1:20 | 20 |
| Ma | 2015 | CS Survey | Mainland China | Second epidemic wave | Poultry workers | HI | Horse RBC | HI titer ≥ 1:160 | 21 |
| Zhu | 2015 | CS Survey | Mainland China | Second epidemic wave | Poultry workers | HI | Horse RBC | HI titer ≥ 1:80 | 22 |
| Lin | 2016 | CS Survey | Mainland China | Second and third epidemic wave | General population | HI and MN | Horse RBC | HI titer ≥ 1:40 and MN titer ≥ 1:40 | 23 |
| Kelvin | 2016 | P Cohort | Hong Kong | second epidemic wave | LPM poultry workers and pig or cattle slaughterhouse workers | HI | Horse and turkey RBC | HI titer ≥1:160 | 24 |
| Yang | 2016 | P Cohort | Mainland China | Second and third epidemic wave | Poultry workers, swine workers, and general population | HI | Horse RBC | HI titer ≥1:80 | 25 |
| Kong | 2016 | CS Survey | Mainland China | - | Poultry workers | HI | Horse RBC | HI titer ≥ 1:160 | 26 |
| Liu | 2016 | CS Survey | Mainland China | - | General population | HI and MN | Chicken RBC | HI titer ≥ 1:20 | 27 |
| Long | 2016 | CS Survey | Mainland China | - | Poultry workers | HI | Horse RBC | HI titer ≥1:160 | 28 |
| Pan | 2016 | CS Survey | Mainland China | - | Poultry workers | HI | Horse RBC | HI titer ≥ 1:160 | 29 |
| Yang | 2016 | CS Survey | Mainland China | - | Poultry workers | HI | - | HI titer ≥ 1:160 | 30 |
| Wang | 2016 | CS Survey | Mainland China | - | Poultry workers | HI | Horse RBC | HI titer ≥ 1:160 | 31 |
| Wang | 2016 | CS Survey | Mainland China | Second epidemic wave | Poultry workers | HI | Horse RBC | HI titer ≥ 1:160 | 32 |
| Wang | 2016 | CS Survey | Mainland China | First and third epidemic wave | Poultry workers | HI | Horse RBC | HI titer ≥ 1:120 | 33 |
| Wang | 2016 | CS Survey | Mainland China | Before 2013 and first epidemic wave | General population and close contact | HI | Chicken RBC | HI titer ≥ 1:20 | 34 |
| Wen | 2016 | CS Survey | Mainland China | - | Poultry workers | HI | Horse RBC | - | 35 |
| Ye | 2016 | CS Survey | Mainland China | Second epidemic wave | Poultry workers | HI and MN | Horse RBC | HI titer ≥ 1:20 and MN titer ≥ 1:20 | 36 |
| Zhu | 2016 | CS Survey | Mainland China | - | Poultry workers | HI | Horse RBC | HI titer ≥ 1:160 | 37 |
| Xiang | 2017 | P Cohort | Mainland China | First and second epidemic wave | General population, poultry workers, and close contacts | HI and MN | Horse RBC | HI titer ≥ 1:20 and MN titer ≥ 1:20 | 38 |
| Zeng | 2017 | CS Survey | Mainland China | - | Poultry workers | HI and MN | Horse RBC | HI titer ≥ 1:160 | 39 |
| Guo | 2017 | CS Survey | Mainland China | - | Poultry workers | - | - | - | 40 |
| Li | 2017 | CS Survey | Mainland China | - | Poultry workers | HI | Horse RBC | HI titer ≥ 1:160 | 41 |
| Li | 2017 | CS Survey | Mainland China | - | Poultry workers | HI | Horse RBC | - | 42 |
| Luo | 2017 | CS Survey | Mainland China | - | Poultry workers | HI | - | HI titer ≥ 1:160 | 43 |
| Wang | 2017 | CS Survey | Mainland China | - | Poultry workers and general population | HI | - | HI titer ≥ 1:80 | 44 |
| Wang | 2017 | CS Survey | Mainland China | - | Poultry workers | HI | Horse RBC | - | 45 |
| Zhang | 2017 | CS Survey | Mainland China | - | Poultry workers | HI | Horse RBC | HI titer ≥ 1:160 | 46 |
| Zhang | 2017 | P Cohort | Mainland China | Second epidemic wave | Poultry workers, swine workers and general population | HI and MN | Horse RBC | HI titer ≥ 1:40 | 47 |
| Ma | 2018 | P Cohort | Mainland China | - | Poultry workers, swine workers, and general population | HI | Horse RBC | HI titer ≥1:10 and MN titer ≥ 1:80 | 48 |
| Gao | 2018 | CS Survey | Mainland China | - | Poultry workers | HI | Horse RBC | HI titer ≥ 1:160 | 49 |
| He | 2018 | CS Survey | Mainland China | - | Poultry workers | HI | Horse RBC | HI titer ≥ 1:160 | 50 |
| Qi | 2018 | CS Survey | Mainland China | - | Poultry workers | HI | Horse RBC | HI titer ≥ 1:80 | 51 |
| Wang | 2018 | CS Survey | Mainland China | - | Poultry workers | HI | Horse RBC | HI titer ≥ 1:160 | 52 |
| Zhou | 2018 | CS Survey | Mainland China | - | Poultry workers | HI | Horse RBC | - | 53 |
| Zhou | 2018 | CS Survey | Mainland China | - | Poultry workers | HI | - | - | 54 |

* R Survey: Retrospective survey; P Cohort: Prospective cohort; CS-survey: Cross-sectional survey; ‘-’indicates no data available; HI: Hemagglutination inhibition test; MN: Microneutralization test; ELISA: Enzyme-linked immunosorbent assay; RBC: red blood cell.

**Reference**

[1] Bai T, Zhou JF, Shu YL. Serologic study for influenza A (H7N9) among high-risk groups in China. N Engl J Med 2013; 368(24): 2339-40.

[2] Hsieh SM, Huang YS, Chang SY, Lin PH, Chang SC. Serological survey in close contacts with a confirmed case of H7N9 influenza in Taiwan. J Infect 2013; 67(5): 494-5.

[3] Qi X, Qian YH, Bao CJ, et al. Probable person to person transmission of novel avian influenza A (H7N9) virus in Eastern China, 2013: epidemiological investigation. BMJ 2013; 347(3):f4752.

[4] Xu W, Lu L, Shen B, Li J, Xu JQ, Jiang SB. Serological investigation of subclinical influenza A(H7H9) infection among healthcare and non-healthcare workers in Zhejiang Province, China. Clin Infect Dis 2013; 57(6): 919-21.

[5] Chen ZQ, Li KB, Luo L, et al. Detection of avian influenza A (H7N9) virus from live poultry markets in Guangzhou, China: a surveillance report. PLoS One 2014; 9(9): e107266.

[6] Wang X, Fang S, Lu X, et al. Seroprevalence to avian influenza A(H7N9) virus among poultry workers and the general population in southern China: a longitudinal study. Clin Infect Dis 2014; 59:e76–83.

[7] Wu J, Zou LR, Ni HZ, et al. Serologic screenings for H7N9 from three sources among high-risk groups in the early stage of H7N9 circulation in Guangdong Province, China. Virol J 2014; 11(1):184.

[8] Xiong CC, Su ZY, Liu ZH, et al. Serological study of antibodies to influenza A viruses among general population in Wuhan city China. Journal Of Clinical Virology 2014; 61(1): 178-9.

[9] Yang SG, Chen Y, Cui DW, et al. Avian-Origin Influenza A (H7N9) Infection in Influenza A (H7N9)-Affected Areas of China: A Serological Study. J Infect Dis 2014; 209(2): 265-9.

[10] Zhou H, Zheng Y, Wang LF, et al. First serologic study for influenza A (H7N9) virus among veterinarians in Guangdong, China. Journal of clinical virology 2014; 60(2): 182-3.

[11] Lu EJ, Chen YY, Liu JW, et al. Surveillance analysis on the occupational population exposed to avian influenza virus and contamination in market environment of Guangzhou city in 2013. Journal of Medical Pest Control 2014; 30(09): 980-1.

[12] Luo ZF, Chen QJ, Yan JP, et al. Epidemiological survey of the first case of H7N9 avian influenza in Fujian Province. Strait Journal of Preventive Medicine 2014; 20(3):13-14.

[13] Xian X. Epidemiological investigation of influenza A virus infection in poultry and its exposed population. Academy of Military Medical Sciences 2014.

[14] Chen JD, Ma J, White SK, et al. Live poultry market workers are susceptible to both avian and swine influenza viruses, Guangdong Province, China. Vet Microbiol 2015; 181(3-4): 230-5.

[15] Fang CF, Ma MJ, Zhan BD, et al. Nosocomial transmission of avian influenza A (H7N9) virus in China: epidemiological investigation. BMJ 2015; 351:h5765.

[16] He F, Chen EF, Li FD, Wang XY, Wang XX, Lin JF. Human infection and environmental contamination with Avian Influenza A (H7N9) Virus in Zhejiang Province, China: risk trend across the three waves of infection. BMC Public Health 2015; 15(1):931

[17] Ma MJ, Ma GY, Yang XX, et al. Avian Influenza A (H7N9) Virus Antibodies in Close Contacts of Infected Persons, China, 2013-2014. Emerg Infect Dis 2015; 21(4): 709-11.

[18] Yin XC, Tao J, Zhao FL, Rao BZ, Liu HL. Antibodies against influenza A (H7N9) virus among veterinarians in China before 2013. Influenza Other Respir Viruses 2015; 9(1): 38-9.

[19] Chen H, Liu HB, Chen DT. Analysis on surveillance results of influenza and human avian influenza in Daxing District of Beijing. Occupation and Health 2015; 31(19): 2624-7.

[20] Lu L, Shen FJ, Song LL, et al. Survey on antibody level of influenza in natural population of Huangpu District of Shanghai. Occupation and Health 2015; 31(11): 1537-9.

[21] Ma HX, Nie YF, Huang XY, Xu BL. Investigation on the distribution of avian influenza virus in external environment and the infection status in poultry - exposed population in Henan. China Inspection and Quarantine 2015; 25(13): 2220-1.

[22] Zhu JL, Wang FY, Pang ZF, Zhang B, Zhang ZG, Wu XH. Survey on KAP about avian influenza and infection status among poultry workers in Jinhua City of Zhejiang Province. Chinese Journal of Health Education 2015; (11): 1033-6.

[23] Lin YP, Yang ZF, Liang Y, et al. Population seroprevalence of antibody to influenza A(H7N9) virus, Guangzhou, China. BMC Infect Dis 2016; 16(1): 632.

[24] To KKW, Hung IFN, Lui YM, et al. Ongoing transmission of avian influenza A viruses in Hong Kong despite very comprehensive poultry control measures: A prospective seroepidemiology study. J Infect 2016; 72(2): 207-213

[25] Yang P, Ma CN, Cui SJ, et al. Avian influenza A (H7N9) and (H5N1) infections among poultry and swine workers and the general population in Beijing, China, 2013-2015. Sci Rep 2016; 6: 33877.

[26] Kong DF, Yin P, Wang X, et al. Analysis on two epidemic waves of human infection with avian influenza A (H7N9) virus and serological monitoring of occupational workers exposed to poultry in Shenzhen City. Occupation and Health 2016; 32(14): 1897-9.

[27] Liu SL. Analysis of the Diversity of Low Pathogenic Avian Influenza Virus in Hubei Province and Serological Analysis of Avian Influenza Virus in Urban Population; University of Chinese Academy of Sciences 2016.

[28] Long MG, Zhang JY, Xiong XP. Monitoring and analysis of poultry occupational exposure population and external environment avian influenza virus in Zhuzhou City in 2014-2015.  Practical Preventive Medicine 2016; 23(07): 854-6.

[29] Pan YZ, Zhao XC, Yuan CY, Wu H, Qi R, Chen C. Surveillance analysis on avian influenza among occupational exposure population and on avian virus from 2013 to 2015 in Wujin of Changzhou. Modern Preventive Medicine 2016; 43(08): 1384-7.

[30] Tang HP, Chen ZH, Huang WW, Zhang TY, Ma JM. Surveillance analysis of serological and environmental avian influenza in occupational exposure population in Longquanyi District of Chengdu City in 2013 and 215. China Health Care Nutrition 2016; (3): 349-50.

[31] Wang L, Pahati N, Chao XF, et al. Live poultry market environment and avian influenza infection status of occupational exposure population in Kashi Area of Xinjiang. Occupation and Health 2016; 32(01): 121-3.

[32] Wang XY, Mei YF, Cui L, Liu HB. Investigation on antibody level of avian influenza environmental monitoring and occupational exposure in Shiyan City. Zhejiang Journal of Preventive Medicine 2016; 28(01): 55-7.

[33] Wang XL, Rao GQ, Cheng XL, Tang YH, Shu J. Monitoring and analysis of occupational exposure population and external environment H7N9 avian influenza virus in Wuyi County, Zhejiang Province, 2013-2014.  Practical Preventive Medicine 2016; 23(06): 712-4.

[34] Wang XC, Wu ZF, Dong XL, et al. Serologic survey of influenza A viruses subtype H7N9, H9, H5, H1N1pdm09, H3 and H1 in rural area of Deqing, Zhejiang. Disease surveillance 2016; (2): 120-5.

[35] Wen QF, Sun XQ, Ma XM, Ma JT, Li T. Serological surveillance and analysis of avian influenza virus in occupational exposure crowd in Ningxia during 2013 - 2015. Chinese Journal of Health Laboratory Technology 2016; 26(24): 3578-81.

[36] Ye XL, Lei YL, Chen YF, Wang XG, Li YM, Chen XY. Serological survey of avian influenza in occupational exposure population in Lishui Zhejiang. Chinese Journal of Health Laboratory Technology 2016; 26(18): 2703-5.

[37] Zhu BL, Huang GH, Mai W, et al. Surveillance of influenza and human avian influenza in Zhaoqing, 2011-2014. China Preventive Medicine 2016; 17(02): 95-8.

[38] Xiang NJ, Bai T, Kang K, et al. Sero-epidemiologic study of influenza A(H7N9) infection among exposed populations, China 2013-2014. Influenza Other Respir Viruses 2017; 11(2): 170-6.

[39] Zeng ZL. Epidemiological Investigation of Avian Influenza Viruses among Natural Environment and Occupational Exposure Population In Jiangxi Province. Nanchang University 2017.

[40] Guo JX, Li HY, Liu YX, Li Z, Zhang YM. Surveillance and analysis of avian influenza in environment and among occupational exposed population in The Sixth Division of Xinjiang Production and Construction Corps, 2013—2016. Bulletin of Disease Control & Prevention 2017; (3): 19-22.

[41] Li BD, Li HY, Zhang H, Yu DS, He J. Avian influenza virus contaminations in environment and Occupational exposure in 2015 and 2016 in Gansu province of China. Chinese Journal of Viral Diseases 2017; 7(04): 267-70.

[42] Li H, Lu NN, Yu J, et al. Monitoring and analysis of serological and environmentally highly pathogenic avian influenza virus in occupational exposure population in Xining City from 2012 to 2015. Shandong Medical Journal 2017; (34).

[43] Luo YH, Zhong Y, Cao ZA. Surveillance of avian influenza virus in Zhuzhou during 2014 - 2016. Chinese Journal of Health Laboratory Technology 2017; 27(21): 3154-6.

[44] Wang QM. Research on level of human infection with influenza A (H7N9) virus in Zhejiang. Ningbo University 2017.

[45] Wang XF, Rong XS, Liu J, Kadil R, Du XY. Analysis of the monitoring results in avian flu occupational exposure group and external environment during 2012 - 2016 in Aksu area. Chinese Journal of Health Laboratory Technology 2017; 27(03): 414-6.

[46] Zhang B, Zhu JL, Zhu SY. Surveillance and analysis of avian influenza virus in population with occupational exposure and out - environment in Jinhua. Chinese Journal of Health Laboratory Technology 2017; 27(02): 235-7.

[47] Zhang XX, Ma CN, Cui SJ, et al. Avian influenza A(H7N9)infection among poultry and swine workers and the general population in Beijing of China during 2013 and 2014. Chinese Journal of Viral Diseases 2017; 7(04): 252-6.

[48] Ma MJ, Zhao T, Chen SH, et al. Avian Influenza A Virus Infection among Workers at Live Poultry Markets, China, 2013-2016. Emerg Infect Dis 2018; 24(7): 1246-56.

[49] Gao ZG, Liu WL, Zhao J, et al. Surveillance of avian influenza in external environment and serological survey of occupational exposure population in Xinjiang in 2016. Occupation and Health 2018; 34(08): 1034-7.

[50] He XS, Li GB, Li B. Surveillance and analysis of poultry occupational exposure population and external environment avian influenza virus in Liangzhou District of Wuwei City. Bulletin of Disease Control & Prevention 2018; 33(02): 21-3.

[51] Qi YP, Gu WL, Fu XF, et al. Contamination status of avian influenza virus in external environment and the levels of H5N6, H7N9 and H9N2 antibodies in poultry-exposed population in Jiaxing City.  Practical Preventive Medicine 2018; 25(04): 417-20.

[52] Wang X, Fang SS, Liu H, et al. Serological investigation of H7N9 avian influenza virus among poultry-exposed workers in Shenzhen City. Occupation and Health 2018; 34(03): 313-6.

[53] Zhou M, Zhang J, Deng SM et al. Surveillance of Avian Flu in the Environment and Occupational Population in Zigong From 2015 to 2017. Journal of Preventive Medicine Information 2018; 34(09): 1232-5.

[54] Zhou X, Wu WL, Hu LH, Li Q. Detection results of avian influenza in environment and occupational exposed population in Panzhihua City from 2015-2017. Occupation and Health 2018; (3): 391-4.
