# Supplementary material for: Seroprevalence of H7N9 infection among humans: A systematic review and meta‐analysis
Source: Influenza Other Respir Viruses. 2020 Mar 10;14(5):587–95. doi: 10.1111/irv.12736 (PMC7431636; doi:10.1111/irv.12736)
Supplement: Supplementary file 2 — Table S2 [file IRV-14-587-s002.docx]

Table S2 Results of study quality assessment of included 54 studies*

| **Author** | **Publication year** | **Population based study** | **Time and location provided** | **Sample size ≥100** | **Avian exposure** | **Characteristics mentioned** | **Used HI** | **Used MN** | **Horse RBC used for HI** | **Seropositive cutoff value provided** | **Refer to WHO criteria** | **Total score** |
| --- | --- | --- | --- | --- | --- | --- | --- | --- | --- | --- | --- | --- |
| Bai | 2013 | YES | YES | YES | YES | YES | YES | YES | NO | YES | YES | 9 |
| Szu-Min | 2013 | YES | NO | NO | NO | YES | YES | NO | NO | NO | NP | 3 |
| Qi | 2013 | YES | YES | NO | NO | NO | YES | NO | NO | NO | NP | 3 |
| Xu | 2013 | YES | YES | YES | NO | YES | YES | NO | NO | YES | NO | 6 |
| Chen | 2014 | YES | YES | YES | YES | YES | YES | NO | YES | YES | NO | 8 |
| Wang | 2014 | YES | YES | YES | YES | YES | YES | NO | YES | YES | YES | 9 |
| Wu | 2014 | YES | YES | YES | YES | YES | YES | YES | YES | YES | NO | 9 |
| Xiong | 2014 | YES | NO | YES | NO | NO | YES | NO | NO | YES | NO | 4 |
| Yang | 2014 | YES | YES | YES | NO | NO | YES | NO | NO | YES | NO | 5 |
| Zhou | 2014 | YES | YES | YES | YES | YES | YES | NO | YES | YES | NO | 8 |
| Lu | 2014 | YES | YES | YES | YES | NO | YES | NO | NO | YES | NO | 6 |
| Luo | 2014 | YES | YES | NO | NO | NO | YES | NO | NO | NO | NP | 3 |
| Xia | 2014 | YES | YES | YES | YES | YES | YES | YES | NO | YES | YES | 9 |
| Chen | 2015 | YES | YES | YES | YES | YES | YES | NO | YES | YES | NO | 8 |
| Fang | 2015 | YES | YES | NO | NO | NO | YES | YES | YES | NO | NP | 5 |
| Fan | 2015 | YES | YES | YES | YES | YES | YES | NO | YES | NO | NP | 7 |
| Ma | 2015 | YES | YES | YES | NO | YES | YES | YES | YES | YES | NO | 8 |
| Yin | 2015 | YES | YES | YES | YES | YES | YES | NO | YES | YES | NO | 8 |
| Chen | 2015 | YES | YES | YES | YES | NO | NP | NP | NP | YES | YES | 6 |
| Lu | 2015 | YES | YES | YES | NO | NO | YES | NO | NP | YES | NO | 5 |
| Ma | 2015 | YES | YES | YES | YES | YES | YES | NO | YES | YES | YES | 9 |
| Zhu | 2015 | YES | YES | YES | YES | YES | YES | NO | YES | YES | NO | 8 |
| Lin | 2016 | YES | YES | YES | NO | YES | YES | YES | YES | YES | YES | 9 |
| Kelvin | 2016 | YES | YES | NO | YES | YES | YES | NO | YES | YES | YES | 8 |
| Yang | 2016 | YES | YES | YES | YES | YES | YES | NO | YES | YES | NO | 8 |
| Kong | 2016 | YES | YES | YES | YES | NO | YES | NO | YES | YES | YES | 8 |
| Liu | 2016 | YES | YES | YES | NO | YES | YES | YES | NO | YES | NO | 7 |
| Long | 2016 | YES | YES | YES | YES | YES | YES | NO | YES | YES | YES | 9 |
| Pan | 2016 | YES | YES | YES | YES | NO | YES | NO | YES | YES | YES | 8 |
| Yang | 2016 | YES | YES | YES | YES | YES | YES | NO | NP | YES | YES | 8 |
| Wang | 2016 | YES | YES | YES | YES | NO | YES | NO | YES | YES | YES | 8 |
| Wang | 2016 | YES | YES | YES | YES | NO | YES | NO | YES | YES | YES | 8 |
| Wang | 2016 | YES | YES | NO | YES | NO | YES | NO | YES | YES | NO | 6 |
| Wang | 2016 | YES | YES | YES | NO | NO | YES | NO | NO | YES | NO | 5 |
| Wen | 2016 | YES | YES | YES | YES | NO | YES | NO | YES | NO | NP | 6 |
| Ye | 2016 | YES | YES | YES | YES | YES | YES | YES | YES | YES | YES | 10 |
| Zhu | 2016 | YES | YES | YES | YES | NO | YES | NO | YES | YES | YES | 8 |
| Xiang | 2017 | YES | YES | YES | YES | YES | YES | YES | YES | YES | YES | 10 |
| Zeng | 2017 | YES | YES | YES | YES | YES | YES | YES | YES | YES | YES | 10 |
| Guo | 2017 | YES | YES | YES | YES | NO | NP | NP | NP | NO | NP | 4 |
| Li | 2017 | YES | YES | YES | YES | NO | YES | NO | YES | YES | YES | 8 |
| Li | 2017 | YES | YES | YES | YES | YES | YES | NO | YES | NO | NP | 7 |
| Luo | 2017 | YES | YES | YES | YES | NO | YES | NO | NP | YES | YES | 7 |
| Wang | 2017 | YES | YES | YES | YES | YES | YES | NO | NP | YES | NO | 7 |
| Wang | 2017 | YES | YES | YES | YES | NO | YES | NO | YES | NO | NP | 6 |
| Zhang | 2017 | YES | YES | NO | YES | NO | YES | NO | YES | YES | YES | 7 |
| Zhang | 2017 | YES | YES | YES | YES | YES | YES | NO | YES | YES | NO | 8 |
| Ma | 2018 | YES | YES | YES | YES | YES | YES | YES | YES | YES | NO | 9 |
| Gao | 2018 | YES | YES | YES | YES | YES | YES | NO | YES | YES | YES | 9 |
| He | 2018 | YES | YES | YES | YES | NO | YES | NO | YES | YES | YES | 8 |
| Qi | 2018 | YES | YES | YES | YES | YES | YES | NO | YES | YES | NO | 8 |
| Wang | 2018 | YES | YES | YES | YES | YES | YES | NO | YES | YES | YES | 9 |
| Zhou | 2018 | YES | YES | YES | YES | NO | YES | NO | YES | NO | NP | 6 |
| Zhou | 2018 | YES | YES | NO | YES | YES | YES | NO | NP | NO | NP | 5 |

* NP: Not provided in the study
