# Supplementary material for: Seroprevalence of H7N9 infection among humans: A systematic review and meta‐analysis
Source: Influenza Other Respir Viruses. 2020 Mar 10;14(5):587–95. doi: 10.1111/irv.12736 (PMC7431636; doi:10.1111/irv.12736)
Supplement: Supplementary file 3 — Table S3 [file IRV-14-587-s003.docx]

Table S3 Summary of regional seroprevalence of H7N9 virus in four populations^*^

| Location | Poultry workers | | | | | Swine workers | | | | | Close contacts | | | | | General population | | | | |
| --- | --- | --- | --- | --- | --- | --- | --- | --- | --- | --- | --- | --- | --- | --- | --- | --- | --- | --- | --- | --- |
|  | Ref | n | event | Seroprevalence  (%) | 95%CI | Ref | n | event | Seroprevalence  (%) | 95%CI | Ref | n | event | Seroprevalence  (%) | 95%CI | Ref | n | event | Seroprevalence  (%) | 95%CI |
| Hong Kong | 1 | 75 | 4 | 4.703 | 0.658, 11.182 | - | - | - | - | - | - | - | - | - | - | - | - | - | - | - |
| Taiwan | - | - | - | - | - | - | - | - | - | - | 1 | 14 | 0 | 0.000 | 0.000, 21.531 | - | - | - | - | - |
| Mainland China | - | - | - | - | - | - | - | - | - | - | - | - | - | - | - | - | - | - | - | - |
| Beijing | 3 | 5832 | 18 | 0.141 | 0.000, 0.536 | 3 | 6399 | 1 | 0.000 | 0.000, 0.009 | - | - | - | - | - | 2 | 5911 | 2 | 0.018 | 0.000, 0.088 |
| Shanghai | 1 | 399 | 0 | 0.000 | 0.000, 0.954 | - | - | - | - | - | 1 | 14 | 0 | 0.000 | 0.000, 21.531 | 1 | 414 | 3 | 0.725 | 0.247, 2.109 |
| Zhejiang | 8 | 4002 | 94 | 0.843 | 0.005, 2.519 | - | - | - | - | - | 2 | 52 | 2 | 1.131 | 0.000, 7.310 | 3 | 5879 | 30 | 0. 153 | 0.000, 0.739 |
| Jiangsu | 4 | 3555 | 16 | 0.267 | 0.037, 0.640 | 1 | 2026 | 2 | 0.056 | 0.000, 0.254 | 2 | 268 | 22 | 7.136 | 4.227, 10.661 | 2 | 3601 | 4 | 0.060 | 0.000, 0.202 |
| Guangdong | 9 | 4729 | 182 | 1.848 | 0.210, 4.646 | 1 | 171 | 2 | 1.170 | 0.321, 4.164 | 1 | 125 | 4 | 3.200 | 1.251, 7.939 | 3 | 6449 | 5 | 0.031 | 0.000, 0.235 |
| Hubei | 2 | 140 | 2 | 0.446 | 0.000, 2.782 | - | - | - | - | - | - | - | - | - | - | 2 | 1886 | 7 | 0.269 | 0.002, 0.811 |
| Gansu | 2 | 1071 | 0 | 0.000 | 0.000, 0.175 | - | - | - | - | - | - | - | - | - | - | - | - | - | - | - |
| Fujian | - | - | - | - | - | - | - | - | - | - | 1 | 25 | 0 | 0.000 | 0.000, 13.319 | - | - | - | - | - |
| Hunan | 3 | 463 | 1 | 0.009 | 0.000, 0.628 | - | - | - | - | - | - | - | - | - | - | - | - | - | - | - |
| Henan | 2 | 1408 | 0 | 0.000 | 0.000, 0.130 | - | - | - | - | - | - | - | - | - | - | 1 | 426 | 0 | 0.000 | 0.000, 0.894 |
| Xinjiang | 4 | 1757 | 0 | 0.000 | 0.000, 0.111 | - | - | - | - | - | - | - | - | - | - | - | - | - | - | - |
| Sichuan | 3 | 425 | 0 | 0.000 | 0.000, 0.521 | - | - | - | - | - | - | - | - | - | - | - | - | - | - | - |
| Anhui | 2 | 434 | 0 | 0.000 | 0.000, 0.413 | - | - | - | - | - | 1 | 103 | 0 | 0.000 | 0.000, 1.113 | - | - | - | - | - |
| Jiangxi | 2 | 825 | 0 | 0.000 | 0.000, 0.000 | - | - | - | - | - | - | - | - | - | - | 1 | 1054 | 0 | 0.000 | 0.000, 0.363 |
| Jilin | 1 | 173 | 0 | 0.000 | 0.000, 2.172 | - | - | - | - | - | 1 | 46 | 0 | 0.000 | 0.000, 3.894 | - | - | - | - | - |
| Shandong | - | - | - | - | - | - | - | - | - | - | 1 | 10 | 0 | 0.000 | 0.000, 16.517 | - | - | - | - | - |
| Hebei | - | - | - | - | - | - | - | - | - | - | 1 | 5 | 0 | 0.000 | 0.000, 31.725 | - | - | - | - | - |
| Guangxi | 1 | 283 | 0 | 0.000 | 0.000, 0.667 | - | - | - | - | - | - | - | - | - | - | - | - | - | - | - |
| Ningxia | 1 | 837 | 0 | 0.000 | 0.000, 0.457 | - | - | - | - | - | - | - | - | - | - | - | - | - | - | - |

^*^‘-’indicates no data available
